# Re-assessing the dimensional structure of the Informant Questionnaire on Cognitive Decline in the Elderly (IQCODE): empirical evidence for a shortened Brazilian version

**DOI:** 10.1186/s12877-015-0098-9

**Published:** 2015-07-31

**Authors:** Michael Reichenheim, Maria Angélica dos Santos Sanchez, Roberto Alves Lourenço

**Affiliations:** Department of Epidemiology – Institute of Social Medicine, Rio de Janeiro State University, Rua São Francisco Xavier, 524, 7° andar/bloco D/sala 7018, 20550-013 Rio de Janeiro, RJ Brazil; Research Laboratory on Human Aging – GeronLab, Internal Medicine Department, Faculty of Medical Sciences, Rio de Janeiro State University, Rua Marechal Rondon 381, 2° andar, 20950-000 Rio de Janeiro, RJ Brazil

**Keywords:** Cognitive impairment, Alzheimer, Epidemiology, Scale validation, Modelling

## Abstract

**Background:**

The dimensional structure, effective number of item responses and item redundancies are controversial features of the Informant Questionnaire on Cognitive Decline in the Elderly (IQCODE) requiring more light. The aims of the present study are to revisit the dimensional structure and propose a shorter version of the instrument.

**Methods:**

The sample comprised 652 elderly and their informants, either attending a geriatric service of a public university clinic or enrolled in a health care provider database in Rio de Janeiro, Brazil. A Confirmatory Factor Analysis (CFA) first tested the originally proposed one-dimensional structure comprised of 26 items. This was followed by sequential Exploratory Structural Equation Model (ESEM) to evaluate alternative models, in particular a bi-dimensional solution. The identification of residual correlations (RC) lead to a shortened 20-item model, which was tested further via CFA.

**Results:**

The original model fitted poorly (RMSEA = 0.073; 90 % CI: 0.069-0.077). Regarding the two-dimensional model, the exploratory procedure (ESEM) indicated several RCs and a lack of factor-based discriminant validity. The ensuing CFA on the one-dimensional model with freely estimated RCs showed an adequate fit (RMSEA = 0.051; 90 % CI: 0.047-0.055). Addressing the identified RCs, the CFA on the abridged 20-item version also showed an adequate fit (RMSEA = 0.058; 90 % CI: 0.053-0.064) and no further RCs.

**Conclusion:**

A one-factor dimensional structure and a reduced version with 20 locally independent items were the most tenable solution. However, although promising, this simpler structure requires further examination before it may be fully supported and recommended.

## Background

By the year 2030, it is estimated that approximately 65.7 million individuals will be affected worldwide by cognitive changes characteristic of dementia. In 2005, the cost associated with this disease was around 315 million dollars a year, and this number has been increasing ever since, causing an enormous impact on societies and families, as well as a strain on public and private health systems [1].

Although a growing body of evidence supports the application of neurochemical diagnostics procedures to detect dementia [2], presently there are no accurate biomarkers based on cheap, non-invasive, and easy to apply techniques for the diagnosis of dementing conditions. Regarded as the leading cause of dementia, Alzheimer's disease has not been yet fully understood; it lacks a clearly defined aetiology and its treatment is entirely based on symptom control. However, the diagnosis in the initial phase may have a number of benefits at the individual and collective levels. Measurement tools for detecting dementia are developing steadily in the context of health services, both to identify new cases and to monitor patients with well-established diagnosis. Nevertheless, evaluations performed in clinical practice are usually based on large and complex neuropsychological tests, which require skilled professionals and whose training is time-consuming and frequently unavailable. Furthermore, these tests have low accuracy under certain conditions, such as in the early stages of dementia, in advanced age, in the presence of acute illness, lack of co-operation or death, severe sensory deficits and/or associated mental disorders, and for screening in populations with low levels of education and literacy [3–5].

Standardized tools based on the informant's report are relevant complementary approaches to the strategies for screening and diagnosing dementia [6, 7]. Originally developed in English for the Australian public, the Informant Questionnaire on Cognitive Decline in the Elderly (IQCODE) is one of the most widely used instruments for rapid application. Designed for use with a relative or close friend living with the elderly for at least ten years, the IQCODE’s perspective is to compare changes in an individual's performance over this time span [8].

The IQCODE has been studied in many countries. Some research focused on the assessment of internal consistency —Cronbach's alpha coefficients ranging from 0.93 to 0.97 [8–14]— and test-retest reliability —*kappa* estimates between 0.75 and 0.96 [9, 15, 16]. Several studies examined different facets of validity, ascertaining both concurrent validity —sensitivities of 69 % to 100 %, specificities of 65 % to 94 %, and areas under the Receiver Operating Curve (AUROC) varying from 0.77 to 0.91 [17–21]— and construct validity —correlations between the IQCODE and cognitive screening tests and neuropsychological tests ranging from −0.12 to −0.78 [17, 22].

As for the dimensional structure of the instrument, most authors suggested a one-dimensional solution, through studies using different statistical methods [8–12, 23, 24]. In 1988, Jorm and Korten [8] tested the dimensionality of the IQCODE for the first time; observing a high correlation between the 26 items presumably dealing with memory or intelligence, and concluded that the instrument was measuring a single dimension of cognitive decline. Shortly after, in 1989, Jorm and Jacomb [9] assessed the factorial composition of the measurement tool through a principal component analysis (PCA) and suggested that the IQCODE was largely measuring a general factor of cognitive decline. Also using PCA, Fuh et al. [10], Morales et al. [12], de Jonghe et al. [11] and Butt [23] reached the same conclusion in the ensuing 20 years. Although their analyses initially suggested underlying multidimensional structures, the authors settled on a single dimensionality since the first factor consistently accounted for most of the common variance.

Using a Rasch Analyses, Tang et al. [24] observed that 22 out of the 26 items (84.6 %) had adequate Infit/Outfit statistics [25], which would reflect the admissibility of the IQCODE as a one-dimensional instrument. In the process, the authors indicated that the high Infit/Outfit values of the remaining four items did not fit the model well and suggested removing them from the set after concluding that they were not closely related to the overall construct. Sikkes et al. [26] also showed that a single dimensional structure would be tenable, despite a high correlation between two hinted factors in their analysis using a graded item response theory (IRT) model.

Despite this apparent agreement on a single dimensionality, one study has raised the possibility of there being more than one factor to represent the construct on cognitive decline [27]. Morales et al. [27] identified two main dimensions explaining nearly 50 % of the total variance, which they coined *memory/learning* and *orientation/operation* following an ancillary qualitative assessment. This emphasize the importance of shedding more light on the instrument's dimensionality, not least because the IQCODE has been undergoing several cross-cultural adaptation processes throughout the years.

Two related issues have yet to be established as well, namely, the effective number of item responses and possible item redundancies. Addressing both would entail shorter versions and therefore improved efficiency.

Regarding the response options, the proposed format containing five levels has been applied since the outset in the majority of the studies [8, 9]. Nevertheless, assessing the adequacy of the number of response options, Tang et al. [24] advocated that redundancies and ambiguities stemming from adjacent categories could be accommodated by merging the responses *much better*, *a little better* and *little change* into a single category, while unifying the options *a little worse* and *much worse* in another. According to these authors, this procedure would also solve the problem of low and sometimes absent endorsements observed in certain categories. Similarly, although without the explicit intention to propose a reduction in the number of response options, Sikkes et al. [26] used a simplified model with three answering levels with the objective to investigate the dimensional structure. They adopted this strategy because in their study, the options *much improved* and *improved* were rarely used. Merging them into one single answering level along with the option *not much change* did not entail any loss of information.

The proposal to reduce the 26-item IQCODE had already been suggested before in some studies [7, 10, 24, 27, 28], although not necessarily involving the exclusion of the same item sets. Table 1 shows the profile of the removed items per study.

**Table 1:** English, Spanish and Brazilian versions of published studies using dimensional assessment and other methods to reduce the number of items of IQCODE

Item		Study based on a dimensional structure (factorial) analysis				
		No		Yes		
		Morales et al.,1995 [27]	Perroco et al.,2009 [28]	Jorm, 1994 [7]	Fuh et al., 1995 [10]	Tang et al., 2004 [13]
		(Corr, ROC)^a^	(ROC)	(PCA)	(PCA, DA)	(RA)
1.	Recognizing the faces of family and friends	X^b^	X	X	X	
2.	Remembering the names of family and friends			X		
3.	Remembering things about family and friends	X				
4.	Remembering things that have happened recently					
5.	Recalling conversations a few days later					
6.	Forgetting what he/she wanted to say in the middle of a conversation			X		
7.	Remembering his/her address and telephone number	X	X			
8.	Remembering what day and month it is					
9.	Remembering where things are usually kept					
10.	Remembering where to find things which have been put in a different place from usual					
11.	Adjusting to any change in his/her day-to-day routine	X	X	X		
12.	Knowing how to work familiar machines around the house	X	X		X	
13.	Learning to use a new gadget or machine around the house					X
14.	Learning new things in general					X
15	Remembering things that happened to him/her when he/she was young		X	X	X	
16	Remembering things he/she learned when he/she was young	X	X	X		
17	Understanding the meaning of unusual words		X	X	X	X
18	Understanding magazine or newspaper articles		X	X	X	X
19	Following a story in a book or on TV		X		X	
20	Composing a letter to friends or for business purposes	X	X	X	X	X
21	Knowing about important historical events of the past	X	X	X		
22	Making decisions on everyday matters				X	
23	Handling money for shopping					
24	Handling financial matters, e.g. the pension, dealing with the bank	X				
25	Handling other everyday arithmetic problems					
26.	Using his/her intelligence to understand what's going on and to reason things through				X	

^a^In brackets, statistical methods used in the studies — Corr: items correlations with total score; ROC: *Receiver Operating Characteristic curve*; PCA: principal component analysis; DA: stepwise discriminant analysis, followed by jack-knife procedure of resampling; RA: Rasch analysis/Item Response Theory modelling

^b^ Removed items

Applying the IQCODE to 257 elderly in a prevalence study of dementia in Spain, Morales et al. [27] explored the correlations between each item score and the total score, proposing thereafter a reduced version with 17 items. To this end, they took the item sets involving the highest correlations, and tested their predictive powers in diagnosing dementia by means of Receiver Operating Curve (ROC) curves. Perroco et al. [28] evaluated 34 patients with Alzheimer' disease and 57 controls, using the IQCODE version with 26 items at the start. Using ROC curves as well, they evaluated each item at a time and proposed withdrawing those showing the smallest AUROC. The process resulted in a 15-items instrument. Notably, both authors resorted to the notion of accuracy, but left aside all issues related to the adequacy of the internal structural properties of the instrument.

Jorm [7] recommended a shortened version by analysing four Australian databases. Using a PCA with varimax rotation, the author preserved the 16 items with the highest loadings. This scale showed good reliability and, as anticipated, high correlations with measures of current cognitive function on one side, and low correlations with measures of pre-morbid cognitive function on the other. Fuh et al. [10] arrived at a subset of 17 items also using a PCA, but the selection process was complemented by a stepwise discriminant analysis, followed by a jack-knife validation procedure. Notably, both studies use an insufficient multivariate model to fully evaluate the instrument’s latent dimensional structure (as the case of PCA [29]), and tend to reach out to functional aspect in support such as internal consistency, external correlations or predictive capability.

Based on 284 informants of elderly admitted for stroke, Tang et al. [24] also reached a 17-item version by employing a Rasch Analysis in this process. The authors used three justifications for removing items to side with a satisfactory evaluation of their reduced instrument’s performance. Two are debatable on the account that they are founded on frail grounds, as the case of evaluating redundancies solely through their semantic contents and/or the high proportion of “*I don’t know*” answers (42 %). The third criteria used to justify removals relied on the aforementioned statistical misfits of some items identified in the Rasch Analysis —e.g., too high Infit/Outfit statistics—, which would be quite sensible, in principle. The problem lies in the assumptions on which an adequate Rasch analysis sustains —one-dimensionality, monotonicity and local (conditional) independence [30, 31]—, but which cannot be exhaustibly ensured in this study. Moreover, despite its sound modelling procedure, this study was limited to a small sample size and confined to patients with acute stroke. In addition, the authors did not test the proposed reduced version following from the removal of redundant items, a step that would have been important to corroborate their findings.

The literature review clearly indicates that there are gaps to be filled in. As seen above, doubts remain in regards to the single dimensionality of the IQCODE, which not only spring from theoretical and conceptual disputes, but also from empirical clues identified in multivariate analyses used for testing the structure. Moreover, there are some issues related to the use of five responses option and to the best-reduced version of the IQCODE, both requiring further examination. One objective of this study was thus to revisit and explore further its dimensional structure. Aiming to offer a shorter version of the instrument, two ancillary objectives of this study were to assess the number of response options, and possible content redundancies between items.

## Methods

### Sample, participants and measurement

The sample consisted of two distinct groups. The first one comprised patients suspected of cognitive disorders, consulted between April and December 2006, in a geriatric service of a public university clinic in Rio de Janeiro, Brazil. The eligible subjects were Brazilian citizens aged 65 years or older evaluated by a comprehensive geriatric assessment in the previous 12 months, and accompanied by an informant aged 23 years or over, familiar of the elders’ daily living activities and cognitive performance over the last ten years. Subjects with psychiatric illness, advanced dementia, severe cognitive and functional impairment, severe motor disability after stroke, and those with severe sensory deficits were excluded. This convenience subsample comprised individuals selected from an evaluation of medical records, and invited to participate when attending a medical follow-up appointments. There were no refusals, and the effective subsample totalled 308 seniors.

The second group, assessed from July 2010 to June 2011, consisted of elderly, clients of a health care provider, also living in Rio de Janeiro, Brazil. They were part of a database of the Rio de Janeiro section of the Frailty in Brazilian Elderly Study (FIBRA-BR) [32]. The same eligibility/exclusion criteria were also applied to this group, except for the need of a previous comprehensive geriatric assessment. Of the 521 elderly initially contacted and responding to the evaluation protocol applied in the first phase of the FIBRA-RJ study, 32 had to be excluded for not meeting the eligibility criteria, and 145 refused to indicate an informant or the informants refused to participate in the study. Therefore, this community sample involved 344 individuals.

The IQCODE version used in the present study was submitted to a cross-cultural adaptation process based on the model proposal of Herdman et al. [33]. A total 169 individuals from a clinical sample participated in the study, of those 35 % were diagnosed with dementia [16]. This study showed a high internal consistency (α = 0.94), as well as a high test-retest agreement estimated on a sub-sample of 97 elderly (ICC = 0.92). The cut-off point identifying ‘optimal’ accuracy was 3.52, showing a sensitivity and specificity of 83.3 % and 80.7 %, respectively [20]. The AUROC was 0.83 [20]. The instrument was also studied in a community sample of 417 individuals and their respective informants. Dementia syndrome was present in 20.4 % of the subjects. In this subset, the best cut-off point was 3.26, entailing a sensitivity of 89 % and a specificity of 72 %. The AUROC curve was 0.88 [21].

In the present study, the Brazilian version of the IQCODE was administered as part of a wider questionnaire covering socio-demographic characteristics of the population. The full content of the 26 items of this version is provided in Sanchez & Lourenço [16] and tables in this article.

### Analysis

The first part of the process consisted in evaluating the relative frequencies of the response options of each item, in order to decide on the number of categories to be used in the ensuing analysis.

The dimensional examination of the IQCODE started by re-assessing the one-factor structure, originally proposed by Jorm & Jacomb [14], using a confirmatory factor analysis (CFA) [34]. Modification indexes (MI) were used to explore possible anomalies. An MI reflects how much the chi-square of the model would reduce if a specific parameter was freely estimated. Expected parameter changes (EPC) indices complement the MIs and project the intensity the parameters would obtain if freely estimated [29]. Exploratory Structural Equation Models (ESEM) were employed next to address the controversy over the number of dimensions (factors) comprising the instrument [35]. These models offer the advantage over the traditional Exploratory Factor Analysis models in that they also allow for assessing other relevant features as, for instance, potential item residual correlations (which may arise from item content redundancies). Rotation is also possible; the current analysis used the *geomin* oblique rotation [36, 37]. MIs and EPCs were also scrutinized.

To refine the findings arising from the exploratory models, the ensuing step consisted of fitting additional confirmatory models, beginning with a two-factor model to assess formally the sustainability of factor-based discriminant validity. To this end, the average variance extracted (AVE) was estimated, which assesses the amount of variance captured by a factor through its manifest items *vis-à-vis* the variance due only to measurement errors [38]. The AVE is a function of the relationship between the item's standardized factorial loadings and their respective errors (*uniqueness*):

$$ {\rho}_{ve}=\left[{\displaystyle {\sum}_{i=1}^k{\lambda_i}^2}\right]/\left[{\displaystyle {\sum}_{i=1}^k{\lambda_i}^2}+{\displaystyle {\sum}_{i=1}^k{\delta}_i}\right] $$

Values range from zero to one. Discriminant factorial validity is supported if, for any given factor, the square root of the AVE is above the correlation of this factor with the others [39], and preferably, without overlapping confidence intervals. In this study, the 95 % CI were estimated using bootstrap (1000 replications) [40, 41].

Item content redundancies were addressed by first inspecting residual correlations through MIs and respective EPCs, and then freely estimating the indicated correlations. These assessments were carried out on the two-factor model fitted to evaluate discriminant validity, as well as on the single factor solution. A reduced model was subsequently explored in the light of the identified redundancies.

Data analysis employed Mplus 7.3 [37]. All models used the Weighted Least Squares Mean and Variance Adjusted (WLSMV) estimator, which fits a probit model on transformed polychoric correlations matrices as required for items with ordinal response options [42]. Model adjustments were evaluated using three indexes. The Root Mean Square Error of Approximation (RMSEA) incorporates a penalty function to deal with the minimum parsimony expressed by the model's degrees of freedom [43]. Values below 0.06 suggest a good fit, while values above 0.10 indicate poor fit, and that the model should be rejected [29, 37]. As measures of incremental fit [29, 43], the Comparative Fit Index (CFI) and Tucker-Lewis Index (TLI) were used to compare the proposed model with a null model of independency. Both indexes range from zero to one, and values greater than 0.95 indicate adequate fit [29]. Theoretical plausibility was also considered in the assessments (e.g., pattern and number of factors).

### Ethical aspects

The Research Ethics Committee of the Pedro Ernesto University Hospital (State University of Rio de Janeiro; process number 1179-CEP/HUPE-CAAE:0054.0.228.000-05) approved the study in conformity with the principles embodied in the declaration of Helsinki. Participants were informed about research procedures and risks before signing an informed consent assuring voluntary participation.

## Results

Of all partaking informants, 79.1 % were women, 76.1 % were the primary caregivers and 63.8 % lived in the same household as the elderly. Their average age was 58 years and the mean education level was 9.9 years (Table 2).

**Table 2:** Socio-demographic characteristics and living arrangements of elderly informants

	Number	Percent^a^	
Sex			
Male	136	20.9	(17.3 – 23.9)
Female	516	79.1	(76.1 – 82.4)
Age (years)			
<29	22	3.4	(2.0 – 4.8)
30 – 39	42	6.4	(4.6 – 8.4)
40 – 49	109	16.7	(14.4 – 19.8)
50 – 59	193	29.6	(25.9 – 33.2)
>60	286	43.9	(39.8 – 47.9)
Educational level (years)			
0	6	9.0	(0.3 – 1.7)
1 – 4	75	11.5	(8.9 – 13.8)
5 – 8	114	17.5	(14.6 – 20.4)
9 – 12	425	65.2	(61.5 – 69.1)
>13	32	4.9	(3.4 – 6.9)
Main caretaker			
Yes	496	76.1	(72.5 – 79.6)
No	156	23.9	(20.4 – 27.5)
Living in the same home			
Yes	416	63.8	(60.0 – 67.5)
No	236	36.2	(32.5 – 40.0)

^a^95 % confidence interval in brackets

The assessment of response levels showed that options *much better* and *a little better* in tandem were endorsed by less than 1 % of the sample in 9 items, 1-2 % in 15 items, and around 4 % in only 2 items. These two categories were thus joined with the adjacent option (*little change*), the subsequent analyses then carried out on 3-level items.

Although the CFI and TLI did not indicate problems (0.968 and 0.965, respectively), the initial CFA involving the original one factor model showed a borderline RMSEA of 0.073 (90 % CI: 0.069-0.077). The MIs and respective EPCs also suggested many features to explore, among those several residual correlations. These findings called for more investigation.

A sequence of Exploratory Structural Equation Models was implemented next. Solutions up to three factors were evaluated. Although the Chi-Square for Difference Test indicated a statistically significant difference between the two and three-factor models, the latter completely lacked clarity as to loading pattern and theoretical interpretation. In turn, the two-factor exploratory model (Model 1, Table 3) suggested a clustering of items 1 to 10 on the first factor and items 11 to 26 on the second factor. All these loadings were statistically significant. Fit indices proved admissible, with a RMSEA of 0.062. However, the correlation of 0.866 between the two factors suggested a lack of discriminant factorial validity. This property was then formally explor ed through a confirmatory model.

**Table 3:** Analysis of the bi-dimensional structure of the Informant Questionnaire on Cognitive Decline in the Elderly (IQCODE) using the exploratory structural equation models (Model 1) and confirmatory factor analysis (Model 2)

Item		Model 1		Model 2	
		factor 1^a^	factor 2^a^	factor 1^a^	factor 2^a^
1.	Recognizing the faces of family and friends	0.678^*^	0.118	0.788^*^	
2.	Remembering the names of family and friends	0.834^*^	−0.004	0.819^*^	
3.	Remembering things about family and friends	0.695^*^	0.197	0.884^*^	
4.	Remembering things that have happened recently	0.892^*^	0.023	0.904^*^	
5.	Recalling conversations a few days later	0.887^*^	−0.003	0.875^*^	
6.	Forgetting what he/she wanted to say in the middle of a conversation	0.699^*^	0.096	0.788^*^	
7.	Remembering his/her address and telephone number	0.708^*^	0.146	0.845^*^	
8.	Remembering what day and month it is	0.637^*^	0.257	0.886^*^	
9.	Remembering where things are usually kept	0.815^*^	−0.008	0.797^*^	
10.	Remembering where to find things which have been put in a different place from usual	0.863^*^	−0.010	0.842^*^	
11.	Adjusting to any change in his/her day-to-day routine	0.200	0.483^*^		0.673^*^
12.	Knowing how to work familiar machines around the house	0.017	0.852^*^		0.865^*^
13.	Learning to use a new gadget or machine around the house	−0.001	0.861^*^		0.857^*^
14.	Learning new things in general	0.185	0.690^*^		0.864^*^
15.	Remembering things that happened to him/her when he/she was young	0.155	0.672^*^		0.817^*^
16.	Remembering things he/she learned when he/she was young	0.075	0.714^*^		0.782^*^
17.	Understanding the meaning of unusual words	0.187	0.621^*^		0.798^*^
18.	Understanding magazine or newspaper articles	−0.083	0.910^*^		0.827^*^
19.	Following a story in a book or on TV	0.030	0.827^*^		0.852^*^
20.	Composing a letter to friends or for business purposes	−0.190	0.925^*^		0.742^*^
21.	Knowing about important historical events of the past	0.136	0.709^*^		0.836^*^
22.	Making decisions on everyday matters	0.074	0.814^*^		0.881^*^
23.	Handling money for shopping	−0.113	1.000^*^		0.932^*^
24.	Handling financial matters, e.g. the pension, dealing with the bank	−0.192	1.000^*^		0.914^*^
25.	Handling other everyday arithmetic problems	0.059	0.852^*^		0.905^*^
26.	Using his/her intelligence to understand what's going on and to reason things through	0.180	0.668^*^		0.837^*^
*f1 ↔ f2* ^b^		0.866^*^ (0.820-0.912)		0.897^*^ (0.875-0.919)	
$\sqrt{\rho_{ve(f1)}}$ ^c^		---		0.844^*^ (0.826-0.862)	
$\sqrt{\rho_{ve(f2)}}$		---		0.840^*^ (0.820-0.859)	
RMSEA^d^		0.062 (0.058-0.067)		0.057 (0.053-0.061)	
CFI^e^		0.979		0.981	
TLI^f^		0.975		0.979	

*p-value <0.001

^a^ Factors loadings

^b^ Correlation factors. 95 % confidence interval estimated using bootstrap (1000 replications)

^c^ Square root of the average variance extracted; 95 % confidence interval estimated using bootstrap (1000 replications)

^d^ Root Mean Square Error of Approximation (RMSEA); 90 % confidence interval in brackets

^e^ Comparative Fit Index (CFI)

^f^ Tucker-Lewis Index (TLI)

Guided by the suggested two factor exploratory configuration, Model 2 in Table 3 shows the factorial discriminant validity assessed via CFA. The fit indexes remained at the same levels, and virtually all loadings stayed above 0.75. Nevertheless, the square root of AVE estimates concerning the two factors — $\sqrt{\rho_{ve(f1)}}$ and $\sqrt{\rho_{ve(f2)}}$ — were lower than the correlation between them, effectively endorsing a low factor-based discriminant validity.

Following the rejection of the two-factor solution, the analysis fell back on the original one-dimensional model, now focusing on the MIs/EPCs in order to examine possible residual correlations. The MIs/EPCs suggested seven residual correlations to be reckoned with, *viz*., iq1↔iq2 = 0.493; iq4↔iq5 = 0.659; iq9↔iq10 = 0.697; iq12↔iq13 = 0.548; iq13↔iq14 = 0.458; iq15↔iq16 = 0,744; and iq23↔iq24 = 0.893. Model 3 of Table 4 displays these residual correlations when freely estimated. Model adjustment remained acceptable, reinforcing the acceptability of the seven residual correlations. The MIs of this model failed to indicate the need to explore additional correlations.

**Table 4:** Confirmatory factor analysis of the unidimensional structure of IQCODE adjusted with residual correlations

Item		Model 3^a^	Model 4^a^
1.	Recognizing the faces of family and friends	0.746^*^	---
2.	Remembering the names of family and friends	0.783^*^	0.785^*^
3.	Remembering things about family and friends	0.860^*^	0.861^*^
4.	Remembering things that have happened recently	0.858^*^	0.860^*^
5.	Recalling conversations a few days later	0.824^*^	---
6.	Forgetting what he/she wanted to say in the middle of a conversation	0.767^*^	0.759^*^
7.	Remembering his/her address and telephone number	0.823^*^	0.825^*^
8.	Remembering what day and month it is	0.862^*^	0.863^*^
9.	Remembering where things are usually kept	0.737^*^	---
10.	Remembering where to find things which have been put in a different place from usual	0.795^*^	0.795^*^
11.	Adjusting to any change in his/her day-to-day routine	0.667^*^	0.667^*^
12.	Knowing how to work familiar machines around the house	0.840^*^	0.833^*^
13.	Learning to use a new gadget or machine around the house	0.818^*^	---
14.	Learning new things in general	0.841^*^	0.842^*^
15.	Remembering things that happened to her/him when she was young	0.773^*^	0.779^*^
16.	Remembering things he/she learned when he/she was young	0.720^*^	---
17.	Understanding the meaning of unusual words	0.791^*^	0.793^*^
18.	Understanding magazine or newspaper articles	0.823^*^	0.825^*^
19.	Following a story in a book or on TV	0.847^*^	0.851^*^
20.	Composing a letter to friends or for business purposes	0.737^*^	0.742^*^
21.	Knowing about important historical events of the past	0.830^*^	0.834^*^
22.	Making decisions on everyday matters	0.878^*^	0.871^*^
23.	Handling money for shopping	0.896^*^	0.896^*^
24.	Handling financial matters, e.g. the pension, dealing with the bank	0.877^*^	---
25.	Handling other everyday arithmetic problems,	0.902^*^	0.900^*^
26.	Using his/her intelligence to understand what's going on and to reason things through	0.829^*^	0.830^*^
iq 01 ↔ iq 02^b^		0.450^*^	---
iq 04 ↔ iq 05		0.537^*^	---
iq 09 ↔ iq 10		0.610^*^	---
iq 12 ↔ iq 13		0.501^*^	---
iq 13 ↔ iq 14		0.432^*^	---
iq 15 ↔ iq 16		0.609^*^	---
iq 23 ↔ iq 24		0.628^*^	---
RMSEA^c^		0.050 (0.046-0.055)	0.058 (0.053-0.064)
CFI^d^		0.985	0.983
TLI^e^		0.983	0.981

*p-value <0.001

^a^ Factors loadings

^b^ Freely estimated residual correlations

^c^ Root Mean Square Error of Approximation (RMSEA); 90 % confidence interval in brackets

^d^ Comparative Fit Index (CFI)

^e^ Tucker-Lewis Index (TLI)

Finally, Model 4 in Table 4 proposes a shortened set of 20 items, based on the assumption of content redundancy identified by the residual correlations. In this tentative configuration, the option was to exclude items with the lowest loading *per* correlated pair, the exception being item 13 that was removed for its involvement in two residual correlations (with iq12 and iq14). The adjustment indices deteriorated somewhat, although still at acceptable levels. Regardless, loadings of the remaining items did not differ much from those found in Model 3. Again, all loadings were statistically significant.

## Discussion

The IQCODE has been extensively studied over the last twenty years. Although studies consistently endorsed the instrument as a tool for screening dementia, gaps in its psychometric history remained open. One of these gaps concerns the dimensionality of the questionnaire. Even though studies tended to support a one-dimensional structure, multi-dimensionality was never completely discarded [27]. Furthermore, studies referred to redundancies between some component items, but were far from a consensus on which should be kept or discarded in any reduced version. Another point still pending concerned the appropriate number of response options *per* item.

A central hypothesis underlying the present study was that the construct of cognitive decline would also be captured through a one-dimensional structure when studied in a different culture from the one where the instrument was conceived. Following suggestions made in the literature, the analysis sequence also touched on a two-dimensional structure. However, a closer examination showed a correlation between the two factors far above the average correlations between the manifest items and their respective factors (assessed by the square roots of the respective average variances extracted). Despite the high factor loadings, which in principle sanction good item discriminant abilities (reliabilities), the solution as a whole showed a negligible discriminant factorial validity. This pointed to the sustainability of a one-dimensional structure, even if other relevant anomalies were still indicating additional problems to address, as for instance, strong residual correlations.

As regards the response options, the findings of the present study mirrored some previous studies as outlined in the Background section. In all items, two options —*much better* and *a little better*—had also very low endorsements, even negligible sometimes (e.g., iq2 and iq4 = 0.61 %). This evidence reinforces that the reduction of response options is an interesting way to improve the adjustment of the items. However, any decision would have to bear a solid theoretical rationale, rather than just the data. From both the point of view of normal neurocognitive aging and the usual evolution of dementia, the two response options seem to be of little relevance, since significant cognitive improvements are rarely expected as people age chronologically and possibly never as neurocognitive disorders evolve.

Regarding the third —and perhaps most important— aspect investigated, the one factor structure CFA (Model 3 in Table 4) effectively identified seven pairs of items involving correlated residuals, thus suggesting violation of local independence assumptions as well as content redundancies from the interpretative stance. As shown in Model 4, the removal of six related items hardly affected the system’s adjustment. This apparent exchangeability of the 26- and 20-item solutions is quite auspicious, since without any loss in content, there is now an operational efficiency gain of almost 25 %.

A mixed pattern emerges on comparing the present findings with the summary provided in the background section concerning items removed in earlier studies [7, 10, 13, 27, 28]. Holding the profile shown in Table 1 against the 13 items containing residual correlations and considered for removal (Model 3 of Table 4), one may note that 8 had been criticised and excluded in previous studies (iq1, iq2, iq12-iq16 and iq22), while five had never been identified up to now (iq4, iq5, iq9, iq10 and i23). In turn, 11 out the 19 items signposted for exclusion in earlier studies failed to show any problem in the present study (iq3, iq6, iq7, iq11, iq17-iq21, iq24 and iq26). As a side note, only two items escaped any criticism thus far (iq8 and iq25).

There is thus little consensus. Still, the authors’ contention is that this picture is not symmetrical. In the present study, the option to deleting items was based on robust empirical evidence, given the residual correlations observed in Model 3 of Table 4 (0.450 to 0.628) are far from trivial. From a substantive viewpoint, there seems to be a theoretical rationale here too, as in the case of the number of item levels. Acknowledging that the answer to an item expresses a manifestation of the latent construct, content redundancies would be accountable on: (1) memories about features (faces and names) regarding relatives and friends (iq1↔iq2 pair); (2) memories on recent events (iq4↔iq5 pair); (3) recalling where some objects are kept and the ability to find them (iq9↔iq10 pair); (4) linking knowledge and learning to handling new appliances and, hence, new things in general (iq12↔iq13↔iq14 triad); (5) remembering things about youth in the (iq15↔iq16 pair); and, finally, (6) the ability of handling one’s own money in the (iq23↔iq24 pair).

As outlined in the Results section, it was decided to remove five items with the smallest loadings (*per* pair), along with item 13 for its involvement in two residual correlations. Nevertheless, the removal of these six items is tentative at best and requires caution. The arbitrary decision to keep items solely on the grounds of higher factor loadings could lead to problems, as this ignores semantic and operational subtleties. Moreover, the preference for a pair or group requires considering its ability to map properly the intensity of the latent construct [25, 44]. Thus, an interesting step forward would be to submit this reduced version to an expert panel and, in the light of the empirical evidences available and the accumulated theoretical background on the construct under scrutiny, decide thereafter which items should effectively be kept or eliminated. New psychometric studies could then be carried out to assess further the new propositions. It is worth emphasizing that, although quite timely in its current 26-item version —around ten minutes interview—, any reduction still enhances efficiency and comes in handy in situations where time pressure is a key factor in selecting measurement tools, such as in large and comprehensive epidemiologic studies or in often busy primary health care (clinical) practices. A 20-item tool, possibly containing items with just three response levels, may be promising from an operational perspective, provided the ability of adequately capturing cognitive decline is preserved.

It would also be interesting to submit versions already reduced by other authors to the same scrutiny proposed here. Smaller and refined new versions may be identified, possibly focusing on other subtleties, such as what to do with the *don’t know* responses [24] or identifying optimal recall periods regarding the component items [45]. Another refinement would be to examine the occurrence of metric and scalar differential item functioning according to the mental state or educational status of the elderly. This is a problem consistently identified in the Mini-Mental State Examination (MMSE) [46–50], which is akin to the IQCODE. Although applied to caregivers rather than the elderly, the latter may also hold group invariance violations, which deserves further investigation.

This study presents some positive aspects. First, the methods are based on procedures proper to analysing latent variables [51], rather than the somewhat feeble data reduction methods such as PCA. Second, the analysis used models and estimators suitable to the polychotomous characteristics of the data at hand (polychoric transformations and probit models via WLSMV estimator). Third, the study was not restricted to the assessment of factor loadings, but also looked into the relationships between factors (assessing the postulated bi-dimensional structure), and presumable local dependencies between items (all models) [34]. Additionally, it is worth mentioning that the effective sample size (n = 652) was quite appropriate. A *post hoc* power study using Monte Carlo simulation, based on the estimates effectively obtained in the one-dimensional 26-items model (Model 3), showed a statistical power above 0.99 [42].

Still, the appraisal of the results requires some caution. Although the Portuguese version used in this analysis was submitted to a careful cross-cultural adaptation process [16, 20, 21], some issues concerning the translation may have affected the response patterns and the present re-inspection of the dimensional structure as a whole. An example would be the addition of a contextualization detail (“*…where did he work when he was young*”) to item 15 of the original version (“*to remember things that happened in his youth…*”). In the specific case, however, this addition seems not to have led to any major problem, since the factor loading proved adequate both in the 26-item model (0.773) and in the shortened 20-items model (0.779).

## Conclusion

Adding to the previous knowledge in the literature, this study tends to reaffirm the IQCODE as an auspicious tool for screening and identifying cases of dementia. However, its use in the original one-dimensional, 26-item format requires caution. While analysable with complex methods to accommodate the significant residual correlations —e.g., structural equation models [43]—, a more efficient version with 20 non-redundant (local independent) items proved quite promising. Yet, this simpler structure requires further examination before it can be fully supported and recommended.

## Abbreviations

AUROC: Area Under the Receiver Operating Curve
AVE: Average Variance Extracted
CFA: Confirmatory Factor Analysis
CFI: Comparative Fit Index
EPC: Expected Parameter Changes
IQCODE: Informant Questionnaire on Cognitive Decline in the Elderly
MI: Modification Indices
PCA: Principal Component Analysis
RC: Residual Correlations
RMSEA: Root Mean Square Error of Approximation
ROC: Receiver Operating Curve
TLI: Tucker-Lewis Index
WLSMV: Weighted Least Squares Mean and Variance (adjusted estimator)
